# Exostosin 1 Knockdown Induces Chemoresistance in MV3 Melanoma Cells by Upregulating JNK and MEK/ERK Signaling

**DOI:** 10.3390/ijms24065452

**Published:** 2023-03-13

**Authors:** Vladlena Pfeifer, Heiko Weber, Yuanyuan Wang, Martin Schlesinger, Christian Gorzelanny, Gerd Bendas

**Affiliations:** 1 Pharmaceutical Department, University of Bonn, An der Immenburg 4, 53121 Bonn, Germany; 2 Department of Dermatology and Venereology, University Medical Center Hamburg-Eppendorf, Martinistr 52, 20246 Hamburg, Germany; 3 Federal Institute for Drugs and Medical Devices (BfArM), 53175 Bonn, Germany

**Keywords:** cancer, chemoresistance, doxorubicin, exostosin1, melanoma, mitoxantrone, HSPG-heparan sulfate proteoglycan

## Abstract

Heparan sulfate proteoglycans (HSPGs) possess various functions driving malignancy of tumors. However, their impact on tumor cell sensitivity to cytotoxic treatment is far less understood. Aiming to investigate this, we depleted HSPGs by downregulating Exostosin 1 (EXT1), a key enzyme in HS formation, or upregulating heparanase in human MV3 human melanoma cells, and investigated their response to cytotoxic drugs. Cytotoxicity of trametinib, doxorubicin, and mitoxantrone was detected by MTT assay. Insights into intracellular signaling was provided by kinome protein profiler array, and selected kinases were inhibited to investigate their impact on cell sensitization and migratory dynamics. EXT1 knockdown (EXT1kd) in MV3 cells affected the activity of doxorubicin and mitoxantrone, significantly increasing EC_50_ values two- or fourfold, respectively. Resistance formation was scarcely related to HSPG deficiency, suggested by enzymatic cleavage of HSPG in control cells. Notably, EXT1kd induced an upregulation of EGFR signaling via JNK and MEK/ERK, and hence blocking these kinases returned resistance to a sensitive level. JNK appeared as a key signal component, also inducing higher migratory activity of EXT1kd cells. Furthermore, EXT1kd upregulated thrombotic properties of MV3 cells, indicated by tissue factor and PAR-1 expression, functionally reflected by a stronger activation of platelet aggregation. EXT1 was confirmed to act as a tumor suppressor, shown here for the first time to affect chemosensitivity of melanoma cells.

## 1. Introduction

Sensitivity of tumors towards cytotoxic treatment is strongly affected by cancer cell communication with the microenvironment [1,2]. In these terms, cell-adhesion-mediated drug resistance is a vital issue, which has been described in its relevance for multiple solid tumor entities [3]. In particular, integrins have been identified as key adhesion receptors attaching the cells at the extracellular matrix (ECM) and thereby inducing a resistance-specific signaling in the cells to functionally antagonize cytotoxic effects [4,5].

Besides integrins, cellular proteoglycans (PGs) have a fundamental role in cellular communication. Heparan sulfates (HS), repeating dimer units of glucuronic acids and glucosamines, are of crucial importance in communication as functional components of PGs, even since they are also constituents of the ECM [6]. As is known, tumor cells display an aberrant expression pattern of HSPG [7] and thus affect multiple processes such as cell growth, invasiveness, or metastasis by binding growth factors, cytokines, coagulation factors, etc., to the HS moieties. For example, the HSPG syndecan 1 has been addressed in several reports and outlined in its pro-tumorigenic functions [8,9].

In an oncological context, appearance and function of HSPGs are far beyond a simple correlation between HSPG activity and a bad prognosis, since HSPGs are essentially required for the homeostasis of several physiological cellular processes. In particular, the role of HSPG in chemosensitivity of tumors has rarely been addressed and appears far from being understood. The balance of pro- and anti-tumorigenic effects of the HSPG network seems not only to be a matter of the HS composition, i.e., sulfation pattern. Size and density of the HSPGs, contributing dominantly to the formation of a cellular glycocalyx, affect their role in oncogenic processes as well. Hence, a strongly reduced glycocalyx by downregulated HSPG biosynthesis has recently been demonstrated to increase metastasis in melanoma cells by attenuating the binding of soluble von Willebrand Factor as a repulsive element in contacting endothelium [10]. 

HSPG synthesis is a multistep process in which HS chains were formed and attached as building blocks to a preformed protein backbone, followed by several modifications, i.e., sulfation, C5-epimerization, or N-deacetylation [11]. In an early phase of HSPG maturation, Exostosin 1 (EXT1), an endoplasmatic residing glycoprotein enzyme, possesses a key control in cellular HSPG formation. As glycosyltransferase, EXT1 together with EXT2 is responsible to elongate the growing HS chain at the protein backbone of PG. To obtain a specific sulfation pattern, this activity is followed by several sulfotransferases and sulfatases in a highly adaptive manner, most likely underlying epigenetic regulations [12]. 

EXT1 and EXT2 have initially been described independently on HSPG synthesis in relation to hereditary multiple exostoses, an autosomal dominant disorder characterized by benign tumors on the active bone growth areas. Since this pathology is triggered by genetic mutations and dysfunction of EXT1 and EXT2, both were considered as potential tumor suppressors [13]. Later, EXT1 and EXT2 have been associated with glycosyltransferase activities [14,15]. However, questions remain as to whether all cellular effects of EXT1 are directly related to HSPG characteristics or based on other intracellular pathways. 

Nevertheless, the fact that EXT1 is epigenetically downregulated in numerous cancer cells supports its estimation as a tumor suppressor. The epigenetic gene silencing of EXT1 by CpG island promoter hypermethylation appears as a common issue in multiple leukemic and non-melanomic skin cancer cells [16], associated with reduced HSPG formation and increased tumor cell proliferation. A recent study reported on EXT1 activity in hepatocarcinoma cells to increase the sensitivity to cytotoxic activities of 5FU/IF-α treatment by inducing higher endoplasmatic stress, leading to autophagy and apoptosis [17]. A study by Liu et al. pointed to the fact that high EXT1 levels act anti-proliferatively, in reference to clinical data of patients with acute lymphocytic leukaemia correlating low EXT1 levels with a bad prognosis [18]. EXT1 knockdown was used as model for HSPG deficiency in melanoma cells and associated with increased metastatic spread [10].

However, although these aspects support the idea that EXT1 acts as a tumor suppressor, other studies detected a supporting role of EXT1 in tumor growth and malignancy. A pro-tumorigenic activity of EXT1 has been reported in a glioblastoma model, since EXT1 knockout reduced cell proliferation in vitro and growth of glioblastoma xenograft in mice by attenuating receptor tyrosine kinase activity [19]. Furthermore, EXT1 was shown to induce stemness in MCF-7 breast cancer cells associated with increased epithelial–mesenchymal transition and doxorubicin resistance, which could be antagonized by EXT1 downregulation [20]. Increased plasma levels of EXT1 were detected as early biomarkers for cholangiocarcinomas in animal models and in humans [21]. Thus, the overall function of EXT1 in oncology is controversially discussed and rather less understood.

Here, we reveal a novel aspect in anti-tumorigenic activity of EXT1. Knockdown of EXT1 in MV3 melanoma cells induces resistance against different cytotoxic agents. Remarkably, resistance is rarely dependent on HSPG deficiency but more associated with intracellular signaling triggered by EXT1. In fact, EXT1 seems to repress a proliferative signaling via the EGFR/JNK/MEK/ERK axis, which is intensified upon EXT1 knockdown and directly linked to resistance. Blocking JNK (c-Jun N-terminal kinase) and ERK (extracellular signal-regulated kinases) in a single or combined manner leads to full sensitization of the cells for cytotoxicity. This is the first report on EXT1 activity in sensitizing melanoma cells for treatment.

## 2. Results

### 2.1. Knockdown of EXT1, Integrin β1, or Overexpressing Heparanase Did Not Impact MV3 Melanoma Cell Proliferation and Adhesion

To figure out the impact of HSPG-driven cell communication on chemosensitivity, we selected the human melanoma cell line MV3 and compared the wild type with three genetic subtypes and their respective controls. Knockdown of EXT1 as a key enzyme in HSPG formation by a shRNA approach displays a strongly attenuated HSPG content, as described previously [10] and exemplarily indicated in Figure S1. Overexpressing human heparanase (HPSE) in MV3 cells (MV3_HPSE_) by transfection with a vector containing full-length cDNA of human heparanase as described previously [22], increases the enzymatic cleavage of HS chains, thus reducing HS chains length. β1 integrins (ITGB1) are key for ECM binding, and HSPG are also considered as co-receptors for certain ITGB1s [23]. Thus, we also performed a lentiviral ITGB1 knockdown approach and used this MV3_ITGB1kd_ variant to focus on the contribution of the ITGB1/HSPG axis. To consider matrix effects, we selected collagen type 1 (COL1) as the most representative ECM component in the context of tumor cell malignancy, i.e., metastasis [24]. 

**Figure 1:**
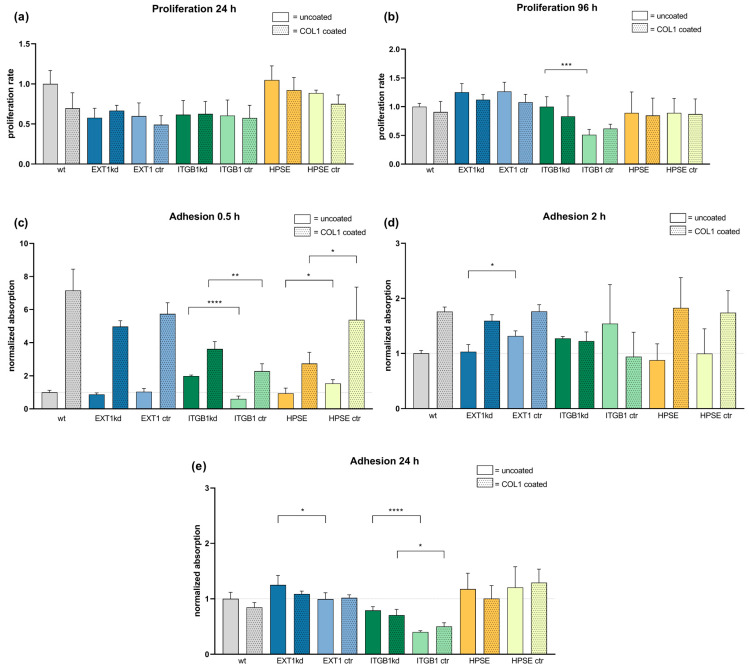
Comparison of cell proliferation rate (**a**,**b**) and cell adhesion capability (**c**–**e**) of MV3 wild-type cells and different transfected MV3 cells versus unspecific control on uncoated or COL1-coated surfaces depending on time. Cell proliferation of the indicated cells was detected by MTT assays over 96 h ((**a**,**b**) and Figure S2) and compared to the proliferation of MV3 wt cells, taken as a rate of 1. (**c**–**e**) Cell adhesion was detected colorimetrically by labeling the cells after thorough washing and fixing at the surfaces. Data indicate that initially a superior binding onto COL1 surfaces after 0.5 h (**c**) is balanced with time after 2 h (**d**) until 24 h (**e**). Data are represented as mean ± SD. For statistics, an unpaired t-test was applied (* = *p* < 0.05, ** = *p* < 0.01, *** = *p* < 0.001, **** = *p* < 0.0001).

To exclude that the genetic modifications affect the cell proliferation rate and thus indirectly the sensitivity to cytotoxic agents, we first checked the impact of genetic variation on cell proliferation vs. their respective controls, taking the proliferation rate of wild-type cells as standard. Furthermore, all cells were compared in their proliferation rate on uncoated as well as COL1-coated surfaces, illustrated in Figure 1a,b for an early and late time range until 96 h. A further data set of cell proliferation is provided in Figure S2. Data indicate that the genetic modifications of MV3 cells do not impact the proliferation rate remarkably, when compared to the respective controls, besides the significantly lower proliferation of the ITGB1 control cells after 96 h. Furthermore, cell binding to COL1 has no impact on proliferation. 

We also checked cell adherence and COL1 binding in a time-dependent manner. Cells were allowed to settle at cultivation surfaces, which were either uncoated or COL1-coated. Cells were labeled and binding was analyzed. In a short time range (0.5 h), COL1 induced a massive increase in cell attachment (Figure 1c), which is balanced at later time points (Figure 1d) and equilibrated after 24 h (Figure 1e). Thus, no differences in cell adhesion can be observed depending on HSPG modification. 

In sum, any changes in chemosensitivity to be considered can neither be induced by deviations in cell proliferation nor in adhesion phenomena.

### 2.2. EXT1 Knockdown Induced a Resistance Formation of MV3 Cells against Doxorubicin and Mitoxantrone

To investigate whether the genetic modification of MV3 cells affect their sensitivity to a cytotoxic treatment, also considering COL1 binding effects, we treated the cells with different cytotoxic agents and detected EC_50_ values by MTT assays, illustrated as logarithmic data (pEC_50_) in the following. Trametinib, a MEK (mitogen-activated protein kinase kinase) inhibitor with therapeutic relevance for melanoma treatment, was compared to the DNA-damaging agents doxorubicin and mitoxantrone as a therapeutic option in late-stage advanced malignant melanoma.

**Figure 2:**
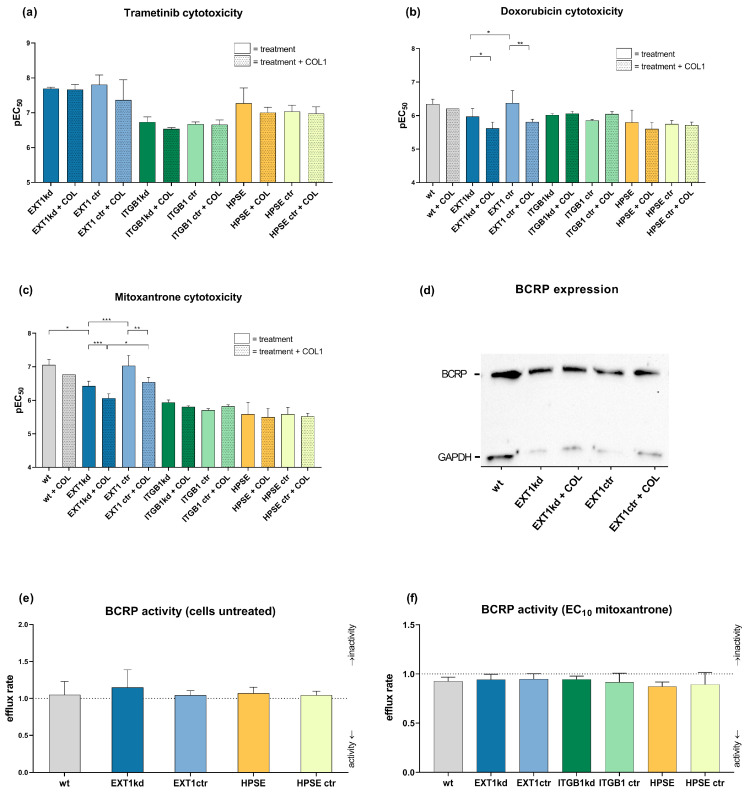
Overview of chemosensitivity (MTT assay) of genetic variants of MV3 cells compared to the corresponding control cell line in relation to COL1 binding. Data were indicated as logarithmic EC_50_ (pEC_50_) values for the treatment with trametinib (**a**), doxorubicin (**b**), or mitoxantrone (**c**). Data indicate that EXT1 knockdown induced a significant resistance formation against doxorubicin and mitoxantrone. (**d**) BCRP transporter expression was detected via Western blot to compare EXT1kd cells and control cells and the impact of COL1. BCRP transporter activity was evaluated by using the pheophorbide assay in the untreated cell variants (**e**) or after stimulating the efflux with mitoxantrone at EC_10_ (**f**). Data are represented as mean ± SD (* = *p* < 0.05, ** = *p* < 0.01, *** = *p* < 0.001). Depicted values are given for at least *n* = 3.

Notably, when comparing the various genetic modifications with their respective controls, neither the genetic status alone nor binding to COL1 had an impact on trametinib sensitivity, shown in Figure 2a, indicated as pEC_50_ values. Solely the controls of the EXT1kd approach showed a twofold higher resistance when cultivated on COL1 (EC_50_ 0.043 µM on COL1 vs. 0.022 µM, respectively).

In contrast, treatment with doxorubicin (Figure 2b) or mitoxantrone (Figure 2c) provided interesting insights. While knockdown of ITGB1 or upregulation of heparanase again showed hardly any impact on sensitivity towards these agents, the EXT1kd approach displayed remarkable effects. On the one hand, COL1 binding reduced the sensitivity to both agents, significantly indicating that loss in EXT1 function, and thus deficiency in HSPG was not critical in contacting COL1. On the other hand, independent from COL1, EXT1kd induced a significant resistance against doxorubicin (EC_50_ 0.46 µM vs. 1.07 µM, for MV3_EXT1ctr_ vs. MV3_EXT1kd_, respectively) (Figure 2b) and against mitoxantrone, showing fourfold higher EC_50_ values (0.088 µM vs. 0.371 µM) (Figure 2c).

Doxorubicin and mitoxantrone are known substrates of ABC efflux transporters, making them susceptible for transporter-induced cell resistance phenomena [25]. To investigate whether EXT1kd is associated with increased efflux transporter activity, we selected breast cancer resistance protein (BCRP) as a common transporter, recognizing both doxorubicin and mitoxantrone. BCRP expression was confirmed by Western blot and did not show detectable differences among the EXT1kd and control cells (Figure 2d). However, to check functional upregulation, we performed a BCRP activity assay using pheophorbide fluorescence. When comparing the intracellular fluorescence of the untreated cells vs. cells treated with a BCRP inhibitor (Ko143) (Figure 2e), no increase in BCRP activity was detected. Under treatment with a non-toxic mitoxantrone concentration (respective EC_10_ values) (Figure 2f), all cells displayed a certain efflux activity, but no differences were visible to explain the resistance in the EXT1kd variant. At this point, we can exclude a vital EXT1/BCRP transporter axis as a probable reason for doxorubicin and mitoxantrone resistance in the EXT1kd cells.

Although the functional basis of EXT1-related resistance remains open, we aimed to elucidate whether the resistance of MV3 cells upon EXT1kd is associated with changes in their HSPG expression (Figure S1). To simulate a probable relationship, we treated MV3_EXT1kd_ and MV3_EXT1ctr_ cells with heparitinase (10 mU/mL) to enzymatically cleave HS chains from the cell surface and investigated the impact on mitoxantrone sensitivity. If deficiency in HSPG function at the cell surface is a reason for resistance formation, MV3 control cells were expected to shift into a more resistant phenotype upon enzymatic treatment. Heparitinase treatment was either performed directly after starting cultivation, after 24 h of mitoxantrone treatment or repeatedly performed every 24 h until reaching the end of the experiments at 96 h for reading out MTT assay. Notably, neither the MV3_EXT1kd_ nor the MV3_EXT1ctr_ cells displayed any deviation in the EC_50_ values in dependence on the enzymatic activity of the different treatment protocols. 

Despite certain limitations when comparing a genetic knockdown of HS synthesis with extracellular HS cleavage, these data refer to the fact that HS cell surface expression, if at all, is only in part responsible for the indicated resistance phenomena.

### 2.3. EXT1kd Induced Higher Coagulability and PAR-1 Expression as Metastatic Markers but Did Not Directly Refer to Resistance

**Figure 3:**
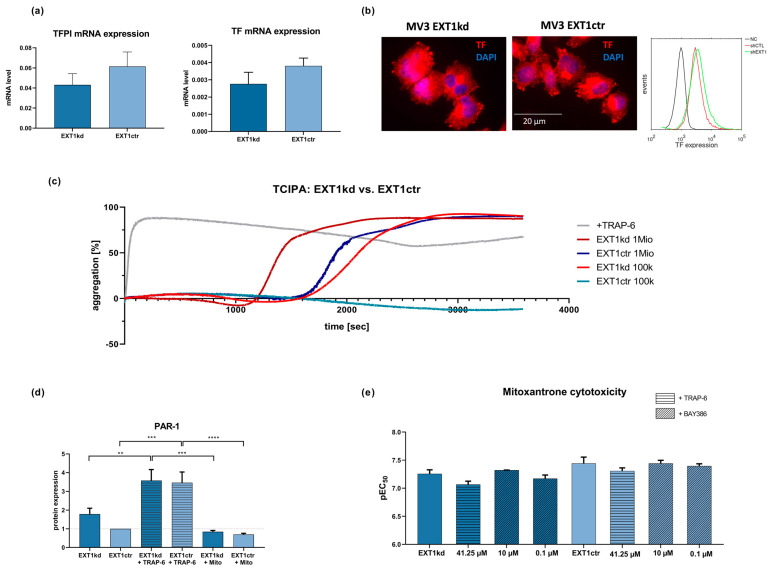
(**a**) qPCR data of TF and TFPI in MV3_EXT1kd_ and MV3_EXT1ctr_ cells. Data indicate that transcriptional expression of TF was very low and appeared not to be significantly affected, while TFPI mRNA levels were downregulated in MV3_EXT1kd_ cells. (**b**) Fluorescence microscopy images of immune stained MV3 cells indicated reasonable protein levels of TF. Moreover, flow cytometric detection of TF expression of MV3_EXT1kd_ and MV3_EXT1ctr_ cells indicated comparably more TF on the surface of MV3_EXT1kd_ cells. (**c**) Detection of platelet aggregation induced by MV3_EXT1kd_ and MV3_EXT1ctr_ cells was given by light transmission aggregometry, using two different cell counts. Data, representative curves of at least *n* = 3, confirmed that EXT1 kd led to a more rapid platelet activation compared to control cells. (**d**) Detection of PAR-1 expression on MV3_EXT1kd_ and MV3_EXT1ctr_ cells by Western blot refers to higher PAR-1 levels in the EXT1 knockdown cells. (**e**) Impact of activating or antagonizing PAR-1 in MV3_EXT1kd_ and MV3_EXT1ctr_ cells on sensitivity to mitoxantrone cytotoxicity. (**a**,**d**,**e**) Data represented as mean ± SD (**d**,**e**). For statistics, an ANOVA was performed (** = *p* < 0.01, *** = *p* < 0.001, **** = *p* < 0.0001).

Previous data suggested that loss of HS at the cell surface is directly associated with an enhanced ability of the cells to induce coagulation [26]. The underlying mechanism is the loss of HS-bound anti-thrombin and tissue factor pathway inhibitor (TFPI) as well as an increased expression of tissue factor (TF), which altogether promotes the formation of thrombin. Therefore, to further understand whether the loss of HS at the surface of MV3_EXT1kd_ is not only linked to an increased resistance towards doxorubicin and mitoxantrone but also enhances a procoagulatory phenotype, we performed corresponding experiments and analyzed the expression of TF and TFPI on MV3 cells using quantitative real-time PCR (Figure 3a). Although the mRNA level of TF was not increased upon EXT1 knockdown, the lower level of TFPI referred to higher TF activity. At the protein level, the EXT1 kd cells displayed slightly higher TF expression when considering immunofluorescence labeling for microscopic detection or detection by flow cytometry (Figure 3b). 

To follow this assumption of increased coagulability, we analyzed platelet activation after contacting MV3_EXT1kd_ and MV3_EXT1ctr_ cells with human platelets. Platelet activation, strongly induced by thrombin as a downstream product of the TF pathway, is indicated by aggregation, which can be followed by light transmission aggregometry [27]. Thrombin dependency is clearly confirmed by using a thrombin receptor (PAR-1) agonist TRAP-6, considered as a positive control activating protease-activated receptor-1 (PAR-1) on platelets. As illustrated (Figure 3c), the EXT1 knockdown led to a higher activation potential of the MV3 cells compared to the control cells, evident by the shorter lag time until reaching a full platelet aggregation using two different cell concentrations.

Tumor-cell-induced platelet activation (TCIPA) strictly depends on thrombin formation [27,28], raising the question whether thrombin, accelerated in formation by the increased TF activity of EXT1 kd cells, also contributes to tumor cell resistance in MV3_EXT1kd_ cells. Notably, expression of PAR-1 by cancer cells is recognized as a tumor-progressive factor and has also been linked to resistance [29]. Aiming to investigate this, we checked the cellular expression of PAR-1 as relevant thrombin receptor by Western blot. Remarkably, the EXT1 kd cells displayed a roughly 40% higher expression of PAR-1 when compared to the control cells (Figure 3d). Interestingly, cell treatment with TRAP-6 further induced PAR-1 expression in both cell lines, suggesting the thrombin/PAR-1 axis as a highly active and adaptive regulatory system in these cells.

To investigate whether PAR-1, upregulated in a procoagulatory scenario, affects cell sensitivity towards mitoxantrone, we preincubated the cells with the PAR-1 inhibitor BAY386, or with TRAP-6 as a PAR-1 agonist (Figure 3e). 

In control cells, TRAP-6 induced a slight shift to higher EC_50_ values (lower pEC_50_), while blocking PAR-1 had no impact on sensitivity to mitoxantrone. This indicated that PAR-1 had no clear impact on the behavior of the control cells. 

In contrast, TRAP-6 mediated a stronger shift to higher EC_50_ values in the EXT1kd cells (Figure 3e, left bars), while blocking PAR-1 at the higher BAY386 concentration sensitized the cells slightly more than in untreated status. Although these findings did not reach significance, they implied a certain contribution of PAR-1 activity to resistance formation. Nevertheless, these findings are in full agreement to our recent study showing higher metastatic capability of MV3_EXT1kd_ cells in mice [10], supporting this finding by a confirmed impact on platelet activation as metastatic trigger.

### 2.4. Insight into Cell Kinome Identified JNK, MEK, and ERK Downstream of EGFR as Targets to Sensitize EXT1kd Cells for Cytotoxic Treatment 

The data above refer to the fact that resistance to doxorubicin and mitoxantrone upon EXT1kdin MV3 cells cannot solely be explained by deregulation of the HSPG pattern or other functional components at the cell surface. This implies that knockdown of EXT1, considered as a tumor suppressor in earlier studies, has additional consequences beyond the synthesis of HS. To follow this, we performed a proteome profiler array focusing on multiple kinases involved in cell signaling and proliferation simultaneously. A full data set, including the effects of COL1, is provided in Figure S3.

**Figure 4:**
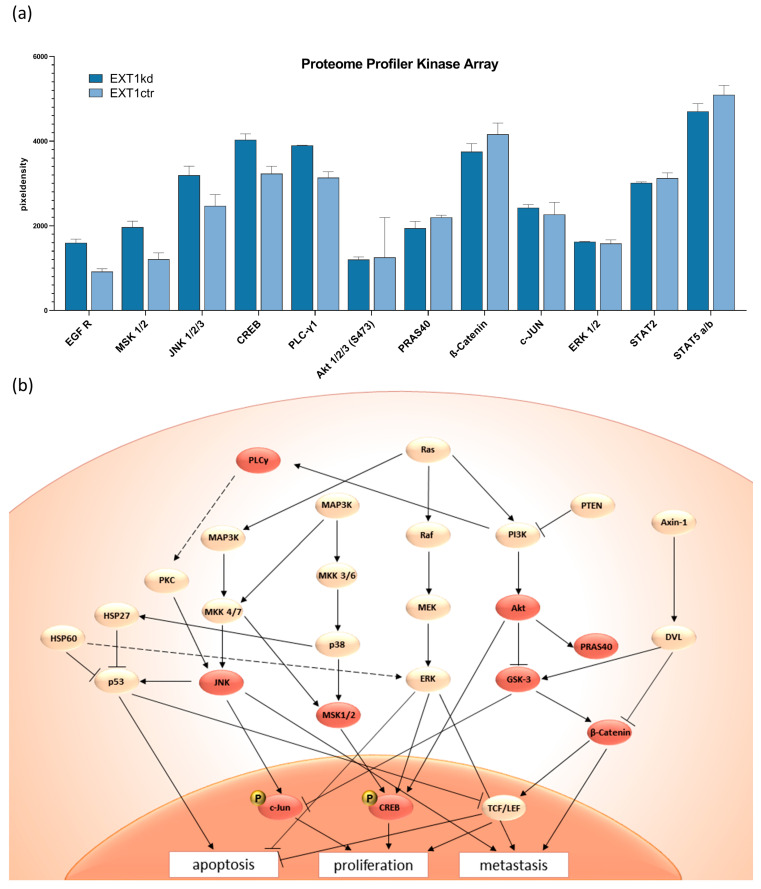
Selected data of a proteome kinase profiler array (**a**) comparing untreated MV3_EXT1kd_ and MV3_EXT1ctr_ cells, given as relative units. (**b**) Scheme of important signaling pathways following EGFR activity and selection for potential interference to affect resistance. Reddish highlighted areas are the deregulated kinases that lead to repressed apoptosis, as well as higher proliferation and metastasis.

A selection of the deregulated kinases (Figure 4a) implies that EXT1kd is associated with an increased EGF receptor (EGFR) activity. Higher c-Jun N-terminal kinases (JNK) and mitogen- and stress-activated kinase (MSK) activities led to activation of c-Jun and cAMP response element-binding protein (CREB), transcription factors associated with intensified cell proliferation. MSK has also been described as a downstream component of MEK/ERK [30]—the latter did not show a deregulation here under the non-activated cell conditions. The findings fit well in signaling cascades, schematically depicted in Figure 4b showing accelerated cell activity and diminished apoptosis for the EXT1kd cells. 

Assuming that the upregulated signaling upon EXT1kd is directly linked to chemoresistance, we examined whether blocking signaling can reverse resistance formation. We selected deregulated key targets and applied tanzisertib and SCH772984 as small molecule inhibitors for JNK, and ERK, respectively. Furthermore, we also applied trametinib to block MEK in this approach. All inhibitors were applied at non-toxic concentrations (Figure S4).

**Figure 5:**
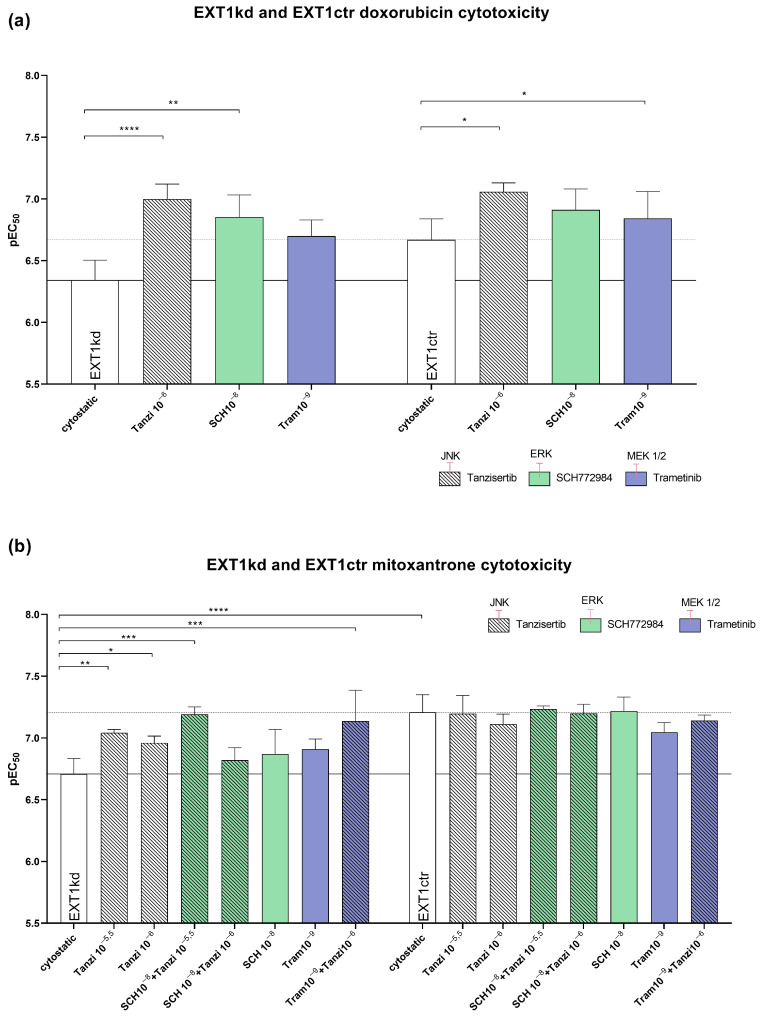
Effect of blocking JNK, ERK, and MEK by using tanzisertib, SCH772984, and trametinib at non-toxic concentrations to sensitize MV3_EXT1kd_ and MV3_EXT1ctr_ cells to cytotoxicity by doxorubicin (**a**) and mitoxantrone (**b**). Cytotoxicity (EC_50_ values) was given by pEC_50_ of at least *n* = 3 for (**a**,**b**). For statistics, an ANOVA was performed (* = *p* < 0.05, ** = *p* < 0.01, *** = *p* < 0.001, **** = *p* < 0.0001). Tanzi = tanzisertib; SCH = SCH772984; Tram = trametinib. Exponents indicate the concentration used of inhibitor in mol.

In the case of doxorubicin treatment, the application of each of the three inhibitors sensitized the EXT1 knockdown cells significantly, reaching a complete sensitization, or even exceeding the sensitivity of the control cells (Figure 5a). The treatment of the control cells with the indicated signaling inhibitors likewise possessed sensitizing effects, significantly for tanzisertib (0.087 µM vs. 0.214 µM; ** *p* = 0.0065).

Next, we investigated whether the indicated inhibitors also affected the sensitivity to mitoxantrone showing a higher resistance rate (Figure 2c) than doxorubicin. While all three inhibitors displayed no effect in sensitizing the control cells (Figure 5b/right bars), the three inhibitors possessed remarkable efficiency to sensitize the EXT1kd cells towards mitoxantrone (Figure 5b/left bars). However, the sensitization level of the control cells was not reached. Notably, blocking JNK by tanzisertib sensitized the EXT1kd cells significantly for mitoxantrone, highlighting JNK inhibition to be more effective than that of MEK or ERK, respectively. To check the effect of simultaneous inhibition of two different signaling downstream pathways (Figure 4b), we next applied a combination of tanzisertib with either SCH772984 or trametinib to block JNK and ERK, or JNK and MEK, simultaneously. Both combinations outperformed the efficiencies of the single components. Remarkably, blocking JNK and ERK led to a full sensitization of the cells for mitoxantrone (EC_50_ 0.062 µM vs. 0.195 µM untreated), which was identical to the control cells.

### 2.5. Blocking JNK Significantly Affected the Motility of EXT1 Knockdown Cells

The above data identified EGFR downstream signaling components as key for sensitizing the MV3_EXT1kd_ cells. Aiming to investigate whether the upregulated signaling axis upon EXT1kd also affects cell dynamics, we performed cell migration studies. Therefore, we applied a 2D migration assay comparing MV3_EXT1kd_ and MV3_EXT1ctr_ cells microscopically over 24 h either untreated or treated with non-toxic concentrations of the ERK inhibitor SCH772984 or the JNK inhibitor tanzisertib. 

**Figure 6:**
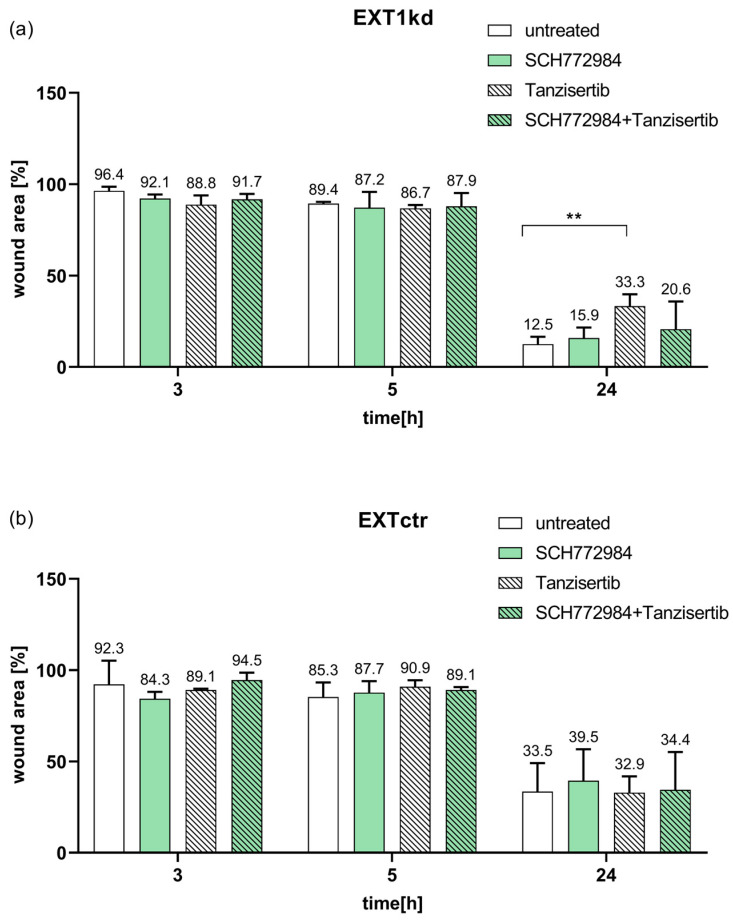
Data of a wound healing assay at 3 h, 5 h, and 24 h of the cell monolayer comparing MV3_EXT1kd_ and MV3_EXT1ctr_ cells either untreated or treated with non-toxic concentrations of the JNK inhibitor tanzisertib or ERK inhibitor SCH772984. Data of wound area were indicated in percent, measured in *n* = 3 for (**a**,**b**) ( ** = *p* < 0.01).

The EXT1kd cells (Figure 6a) showed a certain, not yet significant trend to higher motility, compared the 24 h data with the control cells (Figure 6b) in an untreated state. Notably, dynamics of EXT1kd cells were significantly affected by blocking JNK. Tanzisertib displayed a significant attenuation of cell migration after 24 h. However, blocking ERK, or ERK in combination with JNK inhibition, had a lower effect. In contrast, when affecting JNK and ERK activity in control cells (Figure 6b), no inhibitory effects, aside from a slight decrease in migration by the ERK inhibitor at 24 h, were evident. 

Together, these data highlight JNK as a target to affect increased cell motility and resistance in EXT1 knockdown cells. 

## 3. Discussion

HSPGs possess a key role in tumor cell malignancy, and families of HSPGs, such as syndecans, glypicans, or perlecans, are often upregulated or overexpressed in cancer, associated with growth, proliferation, and a bad prognosis. HS share many characteristics with other glycosaminoglycans (GAGs), representing hydrophilic, linear, and negatively charged polysaccharides, which foster cancerogenesis, e.g., by binding of chemokines or growth factors and increasing their receptor accessibility [31,32]. Although the aberrant expression profiles and metabolic turnover of HSPG or GAGs in cancer is not fully understood, several therapeutic approaches in oncology appear likely to be based on this. Recently, the analysis of soluble GAGs as valid metabolic liquid marker profiles has been developed and outlined for detecting early stages of cancer [33]. These GAGome analyses appear highly promising for future developments in the field of liquid biopsies. On the other hand, a competitive inhibition of cancer HS moieties by similarly structured drugs, i.e., heparin, is an accepted approach to antagonize several tumorigenic pathways [34,35]. Many of the reported anti-cancer activities of heparin derivatives are based on competing with HS-driven processes in cancer [36]. For example, heparin was shown to reduce malignancy in ovarian cancer cells by antagonizing HS of the glypican-3 co-receptor activity at the cell surface, thus attenuating Wnt-signaling and cellular resistance [37]. However, the role of HS in cancer remains versatile and their impact on several aspects in malignancy, such as chemoresistance of cells, is far less investigated. 

To tackle this issue, we genetically affected the cellular HS decoration in human MV3 melanoma cells and investigated the cellular response to cytotoxic treatment with the MEK-inhibitor trametinib, the guideline-based drug in melanoma, or the DNA interfering agents doxorubicin and mitoxantrone, as relevant agents in melanoma therapy. Notably, a massive reduction in cellular HSPG formation by knockdown of EXT1, a key glycosyltransferase in HSPG synthesis, induced a significant increase in EC_50_ values for doxorubicin and mitoxantrone. However, these data give cause to assume that resistance is not or is not only caused by the HSPG deficiency, since two other approaches, the genetic overexpression of heparanase in MV3 cells or the treatment of control MV3 cells by heparitinase, did not shift the cells into a resistant phenotype. This opens the view on HSPG in cancer to differentiate the commonly considered HS effects at the cell surface from the less considered intracellular players and signaling pathways in HSPG formation. 

Here, we expect that the indicated resistance upon EXT1kd is mediated by EXT1 activity beyond its enzymatic function in elongating HS chains, or by deficient HSPG activity at the cell surface. Only a few studies exist addressing EXT1 in oncology, and data refer to either pro- [20] or anti-tumorigenic activities [17]. Furthermore, differentiation between HSPG-mediated effects or intrinsic EXT1 activities can hardly be derived from these findings. We show that loss in EXT1 upregulates a signaling profile that can certainly be associated with increased malignancy. This is partly in agreement with recent findings by Liu et al., who revealed upregulation of ERK upon EXT1 deficiency in ALL [18], although ERK was not deregulated in our assay. JNK was outlined as a key signaling component here, which is in agreement with several considerations of JNK in oncology [38]. The JNK inhibitor tanzisertib appeared highly efficient in inducing a strong sensitization and even full sensitization when combined with an ERK inhibitor. In addition, combined inhibition of JNK and ERK attenuated increased mobility of the EXT1kd cells. The latter fact implies that EXT1 appears as a vital tumor suppressor, not only repressing pro-tumorigenic signaling but also affecting cell mobility. The same is true for the indicated increase in coagulability of the EXT1kd cells, which is a clear factor for increased metastatic spread of these cells, as shown in our recent study [10]. 

Although our present findings highlight EXT1 as a tumor suppressor, shifting cellular HSPG formation indirectly into an anti-tumorigenic context, several aspects remain open and give reason for future studies. Concerning the generalizability of these findings, the relationship of EXT1 and resistance formation is currently derived here from a single melanoma cell line approach. However, preliminary data using other human melanoma cell lines with EXT1kd approaches support these findings and thus overcome this limitation. The fact that the MEK inhibitor trametinib, in contrast to the DNA damaging agents, is not affected in sensitivity upon EXT1kd, can partly be explained by the upregulated signaling. Blocking MEK appears sufficient to equilibrate even an upregulated signaling, thus showing no activity differences between EXT1kd and control cells. However, MEK inhibition supports sensitization against doxorubicin, although full sensitization against mitoxantrone was only achieved in combination with blocking JNK. Nevertheless, the functional, or molecular background of resistance against doxorubicin and mitoxantrone cannot be fully elucidated here. A slight upregulation of BCRP efflux transporter activity might have a certain contribution, but a clear understanding of ABC transporters should be explored in future studies.

Together, our data provide a novel insight into the not yet understood and explored scenario of HSPG and chemoresistance of cancer cells. EXT1, initially having been described as a tumor suppressor before it was referred to as a key enzyme in HS formation and fixed in this role, was shown to suppress a protumorigenic signaling in MV3 melanoma cells with relevance for chemosensitivity, reduced mobility, and reduced coagulability under its activity. These activities are obviously less related to HSPG effects at the cell surface. Highlighting the anti-tumorigenic activities of EXT1, therapeutic prospects arrive from targeting and reversing the epigenetic repression of EXT1 expression, or antagonizing the upregulated signaling upon reduced EXT1 expression, in which JNK appears as promising target. These findings warrant further investigations to scrutinize the potential therapeutic prospects.

## 4. Materials and Methods

### 4.1. Cell Culture

MV3 human melanoma cancer cell line [39] was cultured in RPMI 1640 medium supplemented with 10% FCS, 100 U/mL penicillin, and 100 μg/mL streptomycin (all from PAN Biotech, Aidenbach, Germany). Concerning the transfected MV3 cells, for the downregulation of EXT1, a commercial pool of four EXT1-directed shRNAs (EXT1 Human shRNA Plasmid Kit, CAT#: TL313129, OriGene (Herford, Germany)) has been used, as has recently been described [10], leading to an EXT1 downregulation of approximately 76%. For the upregulation of heparanase, cells were transfected with a vector containing the full length cDNA of human heparanase, as described for other cells [22,40] (cells were a kind gift by Prof. Israel Vlodavsky, Technion, Haifa, Israel). Heparanase upregulation was confirmed by Western blot. 

Downregulation of ITGB1 was achieved by a lentiviral knockdown approach, as described previously [41]. Therefore, cells were seeded in a 96-well plate at a density of 1000 cells/well and let to adhere for 18 h. On the second day, complete cell medium was substituted by 100 μL/well of transduction medium (Polybrene^®^ 4 μg/mL in complete cell medium, Santa Cruz Biotechnology Inc., Heidelberg, Germany). In the next step, the viral particles were thawed and carefully resuspended. To one well, 4 μL of integrin β1 shRNA (h) or control shRNA lentiviral particles were added (sc-35674-V; Santa Cruz). Transfected cells displayed remaining levels of ITGB1 of about 15% compared to unspecific control transfectants. For all transfected cells, 152 µL puromycin (5 mg/mL) was added to the medium. For subcultivation and standard assay use, cells were detached with 0.05% trypsin/0.02% EDTA (PAN Biotech). The absence of mycoplasma contamination was regularly tested.

### 4.2. Adhesion

Coating wells with COL1 (rat tail; Roche Diagnostics GmbH, Mannheim, Germany) was performed at 10 μg/cm^2^, coating volume 30 μL per well of a 96-well plate, as described previously [4]. Plates were placed under laminar air flow at room temperature until complete evaporation of liquid and washed with 50 μL DPBS/well. A total of 30,000 cells were cultured in 160 µL medium on COL1-coated and uncoated surfaces. Non-specific binding sites were saturated with 50 µL of a 1% BSA solution.

After the defined incubation times, each well was aspirated, washed carefully with 100 µL of DPBS, and treated with 50 µL of a paraformaldehyde solution (4%) for 30 min. Staining of the cells was performed with 50 µL methylene blue solution (0.1% in H_2_O). Afterwards, wells were washed three times with 200 µL of deionized water, and 100 µL of HCl solution (0.1 M) was added to dissolve the dye. Absorbance was measured in a Multiskan Ascent microplate reader at a wavelength of 630 nm.

### 4.3. Cytotoxicity Assay

Cell viability assay using MTT 3-(4,5-dimethylthiazol-2-yl)-2,5-diphenyltetrazolium bromide (BioChemica, Applichem GmbH, Darmstadt, Germany) was performed. A total of 3000 MV3 cells were seeded as triplicates at a total volume of 90 µL per well of 96-well plates (Sarstedt AG) either coated with COL1 or left uncoated. After an incubation of 24 h, cells were treated with pathway inhibitors (tanzisertib, SCH772984, or trametinib) (MedChemExpress LLC, Monmouth Junction, Houston, TX, USA) as well as a dilution series of doxorubicin or mitoxantrone (10^−4.5^ to 10^−8.5^ M). After an incubation of 72 h, a MTT solution (20 µL, 5 mg/mL) was added for 1 h at 37 °C and 5% CO_2_. After removing the supernatant, formazan was solubilized in 200 µL of DMSO (Carl Roth GmbH, Karlsruhe, Germany). Plates were analyzed using a plate reader (Thermo Multiscan EX, Thermo, Schwerte, Germany) at 570 nm, with background subtraction at 690 nm. Data were normalized to DPBS as 100% viability.

### 4.4. Proliferation

The proliferation assay is based on the principle of the MTT assay. Cell suspension was seeded into wells and incubated at 37 °C, 5% CO_2_, for different periods of time. The further procedure corresponds to the MTT method. To compare between independently performed experiments, the measured absorbance values of the modified cells were normalized to the wild type.

### 4.5. MTT Assay with Heparitinase

Assays were performed with Heparinase III (H8891; Sigma Aldrich KGaA, St. Louis, Missouri, USA). MV3_EXT1kd_ and MV3_EXT1ctr_ cells were treated with 10 mU/mL at t = 0 h when cells were seeded out, at t = 24 h before starting the cytostatic incubation, and treated repeatedly until assay finalization.

### 4.6. RT-PCR

RNA was extracted using the RNeasy Plus Mini Kit (Qiagen, Hilden, Germany). The concentrations of RNA were measured with a microplate reader (BioteK PowerWave XS2 photometer). The synthesis of cDNA was performed with a Reverse Transcription System (Promega, Madison, WI, USA). The GoTaq^®^ qPCR Master Mix (Promega) system was used for the RT-PCR reaction performed in a real-time PCR system (Light cycler 96 system, Roche). The following primers were used: TFPI- FS: GATGGTCCGAATGGTTTCCAG; TFPI-RS: GATTGCGGAGTCAGGGAGTTA.

### 4.7. Western Blotting

Cell lysis was performed with Invitrogen cell extraction buffer (FNN0011) after washing with DPBS. Whole protein quantification was determined by using BCA protein assay reagents (Pierce™ BCA Protein Assay Kit). SDS-PAGE was performed by using Mini-PROTEAN TGX stain-free Gels from BioRad (Feldkirchen, Germany) after dilution of lysates with Laemmli sample buffer (BioRad #1610747) and denaturation for 30–45 min. PVDF membranes were incubated with respective antibody and mouse anti-GAPDH (Affinity Biosciences; #T0004; 1:10,000 dilution). Western blots were quantified via chemiluminescence using Clarity Western ECL Substrate (BioRad#1705061). Detection was performed with the ChemiDoc XRS+ imaging acquiring system (BioRad) and Image Lab software v. 6.0.1 (BioRad). Antibody Thrombin R (ATAP2) (Santa Cruz biotechnology; sc-13503; 1:200 dilution) was used to detect PAR-1 expression. For BCRP detection, we used ABCG2 antibody (Santa Cruz biotechnology; sc-58222; 1:200 dilution). As a secondary antibody, we used HRP-conjugated anti-mouse IgGκ binding protein (Santa Cruz biotechnology; sc-516102; 1:10,000 dilution).

### 4.8. ABC Transporter Activity Assay

In order to investigate the activity of BCRP, a pheophorbide A (Frontier Scientific Inc., Logan, UT, USA) accumulation assay was performed as described [4]. Cells were cultured and detached by using EDTA and suspended in Krebs–HEPES buffer (10 mM HEPES, 118.6 mM NaCl, 4.7 mM KCl, 1.2 mM KH_2_PO_4_, 4.2 mM NaHCO_3_, 11.7 mM glucose). A total of 40,000 cells were added per well of a 96-well plate. To selectively inhibit BCRP, the inhibitor Ko143 (IC_50_ 0.276 μM, Tocris Bioscience, Bristol, UK) was used at a concentration of 3 μM, and pheophorbide A was used at a concentration of 0.5 μM. After 2 h incubation, fluorescence (λ_ex_ 405 nm, λ_em_ 695 ± 50 nm) was measured by flow cytometry (Guava^©^, Merck). The KHB values were normalized to each inhibited sample to calculate the functional transporter activity as a ratio to maximal inhibition.

### 4.9. Platelet Aggregation Assay

Platelet-rich plasma was obtained from Institute for Experimental Hematology and Transfusion Medicine, University of Bonn, Medical Centre. Platelet separation was performed by centrifugation at 670 × *g*, RT for 10 min, with soft stop. The supernatant was removed, the pellet was resuspended, and the concentration was adjusted to 4 × 10^8^ platelets/mL in platelet buffer (10 mM HEPES, 140 mM NaCl, 3 mM KCl, 0.5 mM MgCl_2_, 5 mM NaHCO_3_, 10 mM glucose). CaCl_2_ at 1 mM final concentration was added to platelet suspensions before use for aggregation.

Tumor-cell-induced platelet aggregation was assessed by LTA using an APACT 4004 aggregometer (Haemochrom Diagnostica GmbH, Essen, Germany). As activating agents, either MV3 cells (1 × 10^4^–1 × 10^6^) or 41.25 μM TRAP-6 (Cayman Chemical, Ann Arbor, MI, USA) were used. Non-activated platelets in platelet buffer correspond to 0% aggregation and 100% aggregation platelet buffer without platelets was measured. Light transmission was measured while the platelet rich buffer was stirred continuously at 1000 rpm at 37 °C in cuvettes.

### 4.10. Proteome Profiler Array

A Proteome Profiler™ human Phospho-Kinase Array kit (R&D Systems GmbH, Wiesbaden-Nordenstadt, Germany; ARY003B) was performed to screen MV3 cells for deregulated kinase signaling pathways. Lysates were prepared according to the manufacturer’s protocol and quantified by BCA Protein Assay Kit (Life Technologies, Thermo Fisher Scientific Inc., Waltham, MA, USA). All array membranes were incubated with 175 μg/mL protein of the lysates and measured according to the protocol.

### 4.11. Cell Migration

A total of 5 × 10 ^5^ MV3 cells were seeded on uncoated 24-well plates (Starlab GmbH, Hamburg, Germany). After 24 h preincubation, wells were washed once with DPBS and fresh FCS-free medium with inhibitor added. Wound healing was observed for various time points with 10-fold magnification using an Axiovert 200 microscope (Zeiss, Jena, Germany).

### 4.12. Immunofluorescence Microscopy

MV3 cells were seeded on coverslips in 24-well plates (Starlab GmbH) overnight. After washing with DPBS, cells were fixed with 4% paraformaldehyde for 10 min at RT. Prior to staining, cells were permeabilized with 0.1% saponin for 15 min. Afterwards, cells were stained with TF antibody (rabbit anti-mouse and human, IgG 1:100, GeneTex GTX01033) for 1 h at RT. After washing with DPBS three times, cells were further stained with the secondary antibody: Alexa 555-conjugated goat anti-rabbit (IgG, Thermo Fisher Scientific, 1:500) for 45 min at RT. Nuclei were stained with DAPI for 20 min at RT. Finally, samples were imaged with a fluorescence microscopy (Observer z.1, Zeiss).

### 4.13. Statistics

Data represent the means and standard error of at least three independent experiments. For statistical analysis, either two-tailed unpaired *t*-test or ANOVA were applied. Statistical significance was indicated with asterisks (* = *p* < 0.05, ** = *p* < 0.01, *** = *p* < 0.001, **** = *p* < 0.0001).

## Data Availability

All the data of this study were presented in the main document or the supplementary files.

## Supplementary Materials

The supporting information can be downloaded at https://www.mdpi.com/article/10.3390/ijms24065452/s1.
