# Supplementary material for: Exostosin 1 Knockdown Induces Chemoresistance in MV3 Melanoma Cells by Upregulating JNK and MEK/ERK Signaling
Source: Int J Mol Sci. 2023 Mar 13;24(6):5452. doi: 10.3390/ijms24065452 (PMC10049486; doi:10.3390/ijms24065452)
Supplement: Supplementary file 1 [file ijms-24-05452-s001.zip › ijms-2187818-supplementary.pdf]

Exostosin 1 Knockdown Induces Chemoresistance in MV3 Melanoma Cells by Upregulating JNK and MEK/ERK Signaling

Vladlena Pfeifer , Heiko Weber , Yuanyuan Wang , Martin Schlesinger , Christian Gorzelanny and Gerd Bendas

Supplementary Figures:

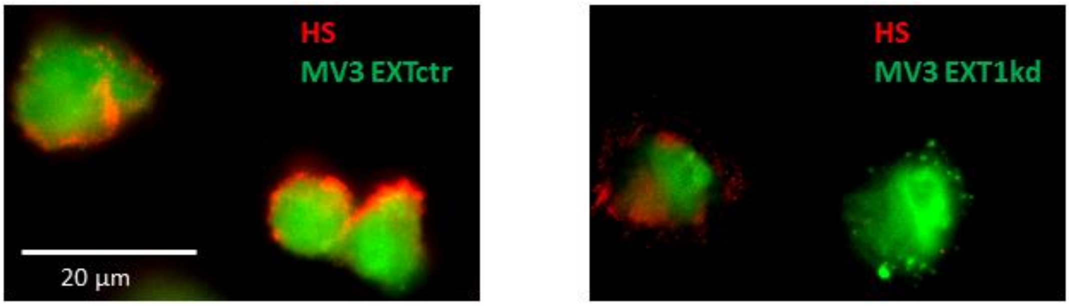

Figure S1 – HS configuration of $MV3_{EXTctr}$ cells and $MV3_{EXT1kd}$ visualized with immunofluorescence microscopy detecting glycans by staining with a ATTO 646 N-conjugated WGA.

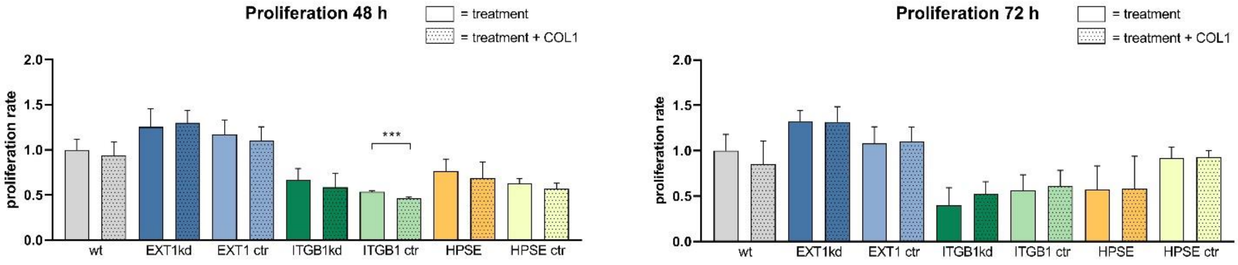

Figure S2 –Cell proliferation rate of MV3 wild type cells and different transfected MV3 cells ($MV3_{EXT1kd}$ and $MV3_{EXTctr}$, $MV3_{ITGB1kd}$ and $MV3_{ITGB1ctr}$, $MV3_{HPSE}$ and $MV3_{HPSEctr}$) versus unspecific control on uncoated or COL1-coated surfaces. Proliferation rate was detected by MTT assay, shown here for 48 h and 72 h in addition to the data given in Figure 1 a and b.

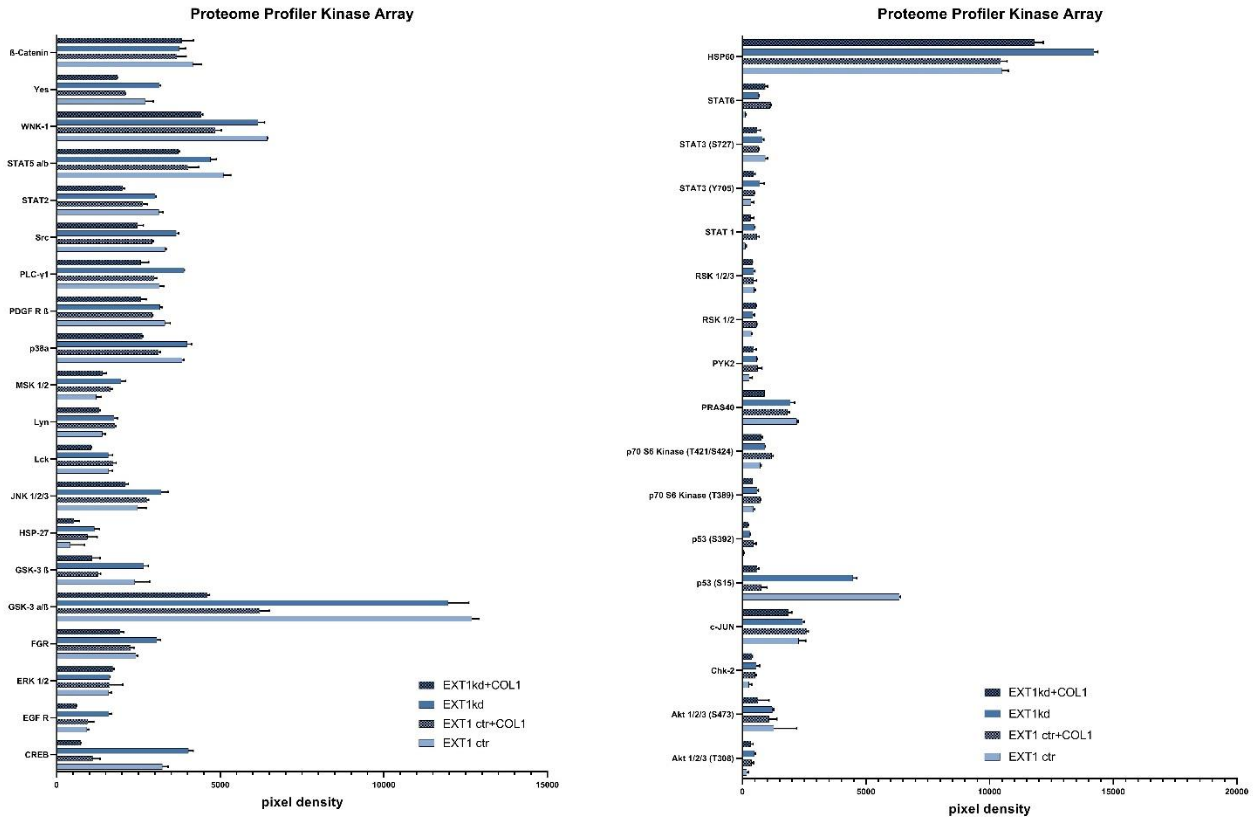

Figure S3 - Proteome Profiler™ human Phospho-Kinase Array was performed with $MV3_{EXT1kd}$ and $MV3_{EXTctr}$ cells. Data depict the relative expression of kinases in $n = 2$.

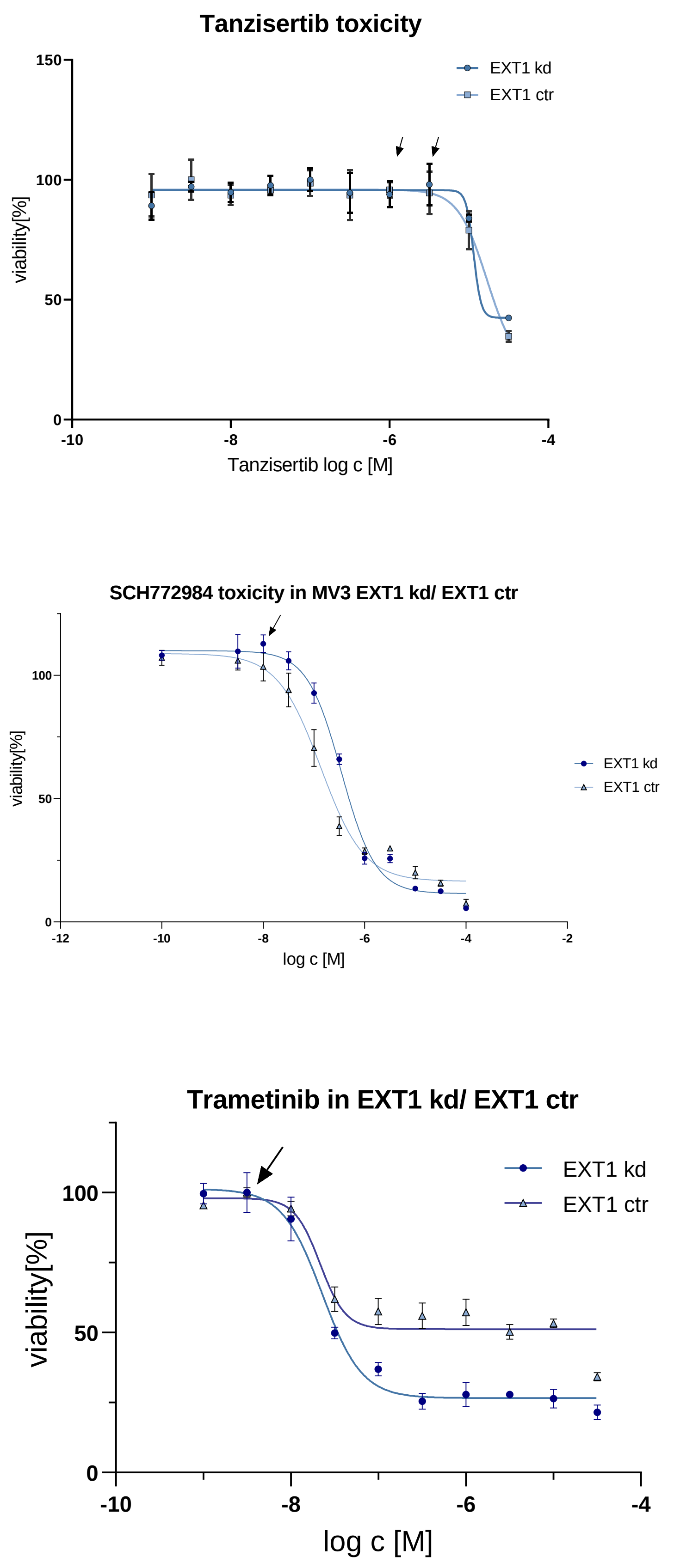

Figure S4 – Cytotoxicity of tanzisertib, SCH772964 and trametinib in $MV3_{EXT1kd}$ and $MV3_{EXTctr}$ cells. Arrows indicate the non-toxic concentration used for the experiments shown in Figure 5 and Figure 6.
